# The Impact of Cheering on Sports Performance: Comparison of Serie A Statistics Before and During COVID-19

**DOI:** 10.7759/cureus.17382

**Published:** 2021-08-23

**Authors:** Alessandro Rovetta, Alessandro Abate

**Affiliations:** 1 Mathematical, Statistical and Epidemiological Models, Technological and Scientific Research, Redeev Srl, Naples, ITA; 2 Mathematical, Statistical and Epidemiological Models, Research and Disclosure Division, Mensana Srls, Brescia, ITA; 3 Massage Physiotherapy, Five Personal Trainer, Brescia, ITA

**Keywords:** sport psychology, covid-19, home advantage, lockdown, audience

## Abstract

Background: The role of cheering in home advantage in sports performance is unclear. As anti-coronavirus disease 2019 (COVID-19) restrictive measures have prevented crowds from entering stadiums, analysis of the past two football seasons can reveal important details.

Objective: This paper aims to compare the last two football seasons in Italy with the previous six, highlighting changes due to the absence of cheering.

Methods: We compared the average percentages of points obtained in home matches from 2013 to 2019 with those in the timelapse 2019-2021. The same operation was performed with referee statistics, such as fouls, penalties, and cards awarded against home teams. To do this, we used Welch's t-test and percentage increases. Pearson and Spearman's correlations were searched between the percentages of points collected in home matches and total points earned from 2013 to 2021.

Results: The average percentage of points collected by teams in home matches dropped by 8% (Welch’s t = −4.3). The negative correlations between home collected points and total points in 2013-2019 timelapse have significantly diminished during the last two seasons (Welch’s t = 6.2), approaching zero. Penalties against home teams have increased by 30% (Welch’s t = 2.6), reaching 51.4%.

Conclusions: This research provides statistical evidence supporting the crowd’s impact on sports and refereeing performance in Serie A. However, our results also suggest that part of the home advantage is linked to factors independent of the audience. Future research can deepen the above phenomena from a theoretical-psychological point of view.

## Introduction

The effect of public cheering on sports performance has been historically investigated by vast scientific literature [1]. The studies range from analyzing the influence of fans on individual athletes to that on teams [2,3]. Nonetheless, researchers do not always come to similar conclusions, for example, while Strauss' survey suggests that spectators' cheering does not encourage better performance from home teams [3], Ponzo and Scoppa showed the existence of a sizable crowd support's effect on the home advantage generated through (i) the encouragement of players and (ii) the positive conditioning of referee decisions [4]. Furthermore, other studies have proposed additional factors that can increase the home advantage, such as away-team travels, familiarity with local facilities, and territoriality or tactics issues [5]. In this context, the coronavirus disease 2019 (COVID-19) pandemic offered an unprecedented historical scenario; indeed, the non-pharmacological countermeasures adopted to contain the infection - such as social distancing and lockdowns - forced teams and athletes to perform without the fans' support for most of the seasons [6]. This situation has given rise to many studies aimed at unraveling the skein of home advantage once and for all [7]. However, even in this case, the results were often discordant with each other; in particular, while Wunderlich et al. found that home advantage remains in European top-class football matches played without spectators [8], Hill and Van Yperen conclude that the home field advantage may indeed be lost when these are absent [9]. At the same time, Sors et al. have obtained results that bring further support to the claim that, among all the variables contributing to home advantage, crowd noise has a relevant role [10]. Benz and Lopez used a bivariate Poisson regression to prove the dependence of the phenomenon on the league under consideration [11], and Tilp and Thaller even found a reversal from home advantage to disadvantage [12]. Therefore, the position of scientists at the international level is far from unambiguous, which requires an intensification of research in this field. We emphasize that the discrepancies mentioned above may depend on the approach adopted to carry out the analysis and the contexts examined. Specifically, (i) the amateur sport could differ substantially from the professional sport when the ability of athletes to respond to external stimuli is concerned [13-15]; (ii) fans' reactions could depend on the sport, season, and even the match or event in question [16]; (iii) the economic condition of the clubs could affect the performance of athletes [17]; (iv) supporters can often express disappointment during a match or sporting performance [2]; and (v) other psychological, social, and cultural variables could unpredictably alter sports performance [18-20]. Since the enormous damage caused by COVID-19 from a psychological point of view falls into the latter category of bias [21,22], it is necessary to interpret all the evidence found with caution. In this regard, the present research aims to evaluate the impact of the stadium supporters on the sporting performance of footballers and referees' decisions in Italy.

## Materials and methods

Data collection

The statistics of the Italian soccer competition *Serie A* were collected from the *Who Scored?* website [23]. All data were reported on Microsoft Excel (v.2021, Microsoft Corporation, Redmond, Washington) and analyzed through the functions integrated into the latter and the XLSTAT (v.2021.2.2, Addinsoft, Paris, France) and Real Statistics (v. June 2021) packages.

Procedure

We analyzed the data from the 2013 to 2014 season up to the 2020 to 2021 season. Specifically, we calculated the percentage of points collected in home games (home game points [HGPs]) per season, the average percentage of completed passes per season, and referee statistics per season, looking for any trends or anomalous oscillations during the above period. In this way, we observed whether unusual behaviors occurred in concomitance of the COVID-19 pandemic.

Data analysis

We performed the Shapiro-Wilk test and a graphical check of frequency histogram and Q-Q plot to assess the normality of each dataset. We found that all datasets were sufficiently normal (Appendix). After that, we calculated the percentage increases (sometimes indicated as Δ%) and Welch's t-test to quantify the intensity and significance of the detected fluctuations in HGPs per season, respectively. For each statistic analyzed, a global t-test was performed to overcome the limited reliability of the multiple comparisons without adopting ANOVA or similar methods. Specifically, after verifying the stationarity of the series through a graphical check, a global t-test compared the 2013-2019 series with the 2019-2021 series. However, the season-season comparison has been maintained as each season can have unique characteristics. In addition, we calculated the Pearson and Spearman correlations between the HGPs and the total points per season. We excluded up to a maximum of two teams (10% of the dataset) from the correlational analysis when these represented outliers. Changes in correlations were also evaluated with the above methods. We verified the presence of possible trends through the Mann-Kendall test and Sen's slope. Two-tailed P-values were adopted as graded measures of the strength of the evidence against the null hypothesis. To ensure the analysis transparency, we reported all P-values alongside their respective quantities.

This analysis assumes that only sports factors such as fans' supporting, referee conditioning, away-team traveling, familiarity with local facilities, and territoriality or tactics issues can drive home advantage. Furthermore, we assumed that, without COVID-19, the 2019-2020 and 2020-2021 seasons would have followed the same distribution as the seasons in the 2013-2019 timelapse.

## Results

Teams

We found evidence supporting a significant reduction in the percentage of points earned by Serie A teams playing home games during COVID-19-affected seasons (∆% = −7.8, Welch’s t = −4.3). In particular, (i) the two minimum values ​​of the average percentage of points collected in home games (HGPs) were achieved in the 2019-2020 and 2020-2021 seasons, and (ii) comparing the 2019-2020 and 2020-2021 seasons with the previous, the smallest discrepancies between HGPs were found between the 2019-2020 and 2020-2021 seasons (Table 1). Conversely, the pandemic does not appear to have affected the variance of the data.

**Table 1:** Average percentage of home points collected by Serie A teams, season by season, from 2013 to 2021. Δ% = percentage increase.

Serie A	2013-2014	2014-2015	2015-2016	2016-2017	2017-2018	2018-2019	2019-2020	2020-2021
Average % home points	61.6	56.5	59.9	60.5	56.5	58.4	53.1	54.5
Standard deviation	8.7	5.2	6.4	6.3	9.8	10.2	5.3	8.8
Mean standard error	2.0	1.2	1.4	1.4	2.2	2.3	1.2	2.0
t-test 2020 vs all	−3.7	−2.1	−3.7	−4.0	−1.4	−2.1	/	−0.6
Δ% 2020 vs all	−13.8	−6.1	−11.4	−12.2	−6.0	−9.1	/	−2.6
t-test 2021 vs all	−2.5	−0.9	−2.2	−2.5	−0.7	−1.3	0.6	/
Δ% 2021 vs all	−11.5	−3.5	−9.0	−9.9	−3.5	−6.6	2.7	/

By performing the Mann-Kendall test on the 2013-2019 series and comparing it with that of the 2013-2021 series, we obtained a 78.4% increase in Sen's slope (from −0.51 to −0.91) and an 86.7% decrease in P-value (from 0.45 to 0.06). This shows how the advent of COVID-19 has drastically lowered HGPs over the last two seasons. Therefore, the stadium audience factor could be a relevant component of the home advantage. In addition to this, the restrictive measures seem to have heavily affected the physiognomy of the competition (Table 2); indeed, the negative correlations between HGPs and total points observed in 2013-2019 timelapse have drastically diminished during the last two seasons (Fisher Z-values average Δ% = −78.9, Welch’s t = 6.2).

**Table 2:** Correlations between the average percentage of home points and total points collected by the Serie A teams, season by season, from 2013 to 2021.

Serie A	2013-2014	2014-2015	2015-2016	2016-2017	2017-2018	2018-2019	2019-2020	2020-2021
Pearson correlation	−0.42	−0.47	−0.39	−0.36	−0.57	−0.54	−0.06	−0.15
P-value	0.065	0.051	0.097	0.114	0.008	0.021	0.803	0.53
Spearman correlation	−0.48	−0.46	−0.34	−0.34	−0.63	−0.45	−0.13	−0.04
P-value	0.032	0.053	0.16	0.139	0.003	0.063	0.588	0.878
Excluded values	0	2	1	0	0	2	0	0

The average percentage of completed passes increased during the pandemic compared to previous seasons (∆% = +2.9, Welch’s t = 3.7; Table 3). Moreover, the variance of the same quantity reached historic lows, testifying that the effect was common to all the teams (Table 3).

**Table 3:** Average percentage of successful passage by Serie A teams, season by season, from 2013 to 2021. Δ% = percentage increase.

Serie A	2013-2014	2014-2015	2015-2016	2016-2017	2017-2018	2018-2019	2019-2020	2020-2021
Average % passes successful	80.5	79.7	78.7	80	80.3	81.1	82.2	82.4
Standard deviation	3.4	3.6	4.8	4.3	4.4	3.9	3.4	3.4
Mean standard error	0.8	0.8	1.1	1	1	0.9	0.8	0.8
t-test 2020 vs all	1.6	2.3	2.7	1.9	1.5	1	0	−0.1
Δ% 2020 vs all	2.1	3.2	4.5	2.8	2.4	1.4	0	−0.2
t-test 2021 vs all	1.7	2.4	2.8	2	1.6	1.1	0.1	0
Δ% 2021 vs all	2.3	3.3	4.7	3	2.5	1.6	0.2	0

Intra-2019 analysis

**Figure 1:**
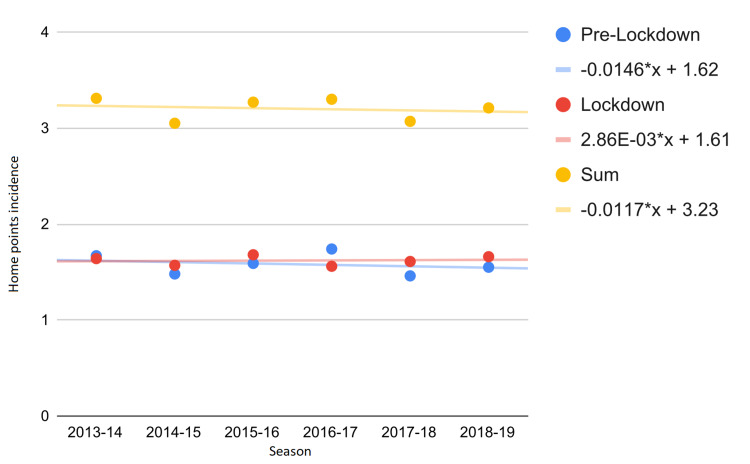
Incidence of home points from 2013 to 2019. The equations of the interpolating lines are shown on the right side.

**Figure 2:**
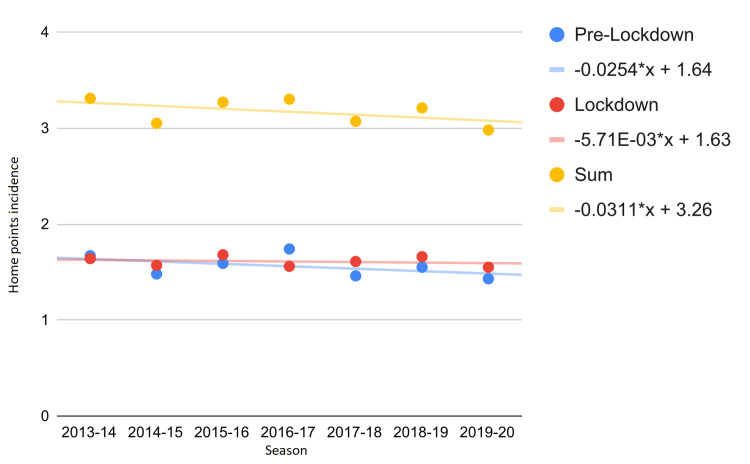
Incidence of home points from 2013 to 2020. The equations of the interpolating lines are shown on the right side.

Since most of the 2019-2020 season was played with the audience, we also analyzed the intra-season impact. In particular, we have divided the season into the pre-lockdown and lockdown periods, analyzing the trend in the incidence of home points from 2013 to 2020. Figures 1, 2 show that, during the first lockdown, the trend of average home points per season was reversed (Δ% = −300.0%). Notwithstanding that such trend was not strong enough to be statistically significant, the change in the Mann-Kendall P-value was considerable (from P = 0.999 to P = 0.548). However, the 2019-2020 season also influenced home points collected before the lockdown period (Δ% = −74.0%, Figures 1, 2).

Leaderboards

**Figure 3:**
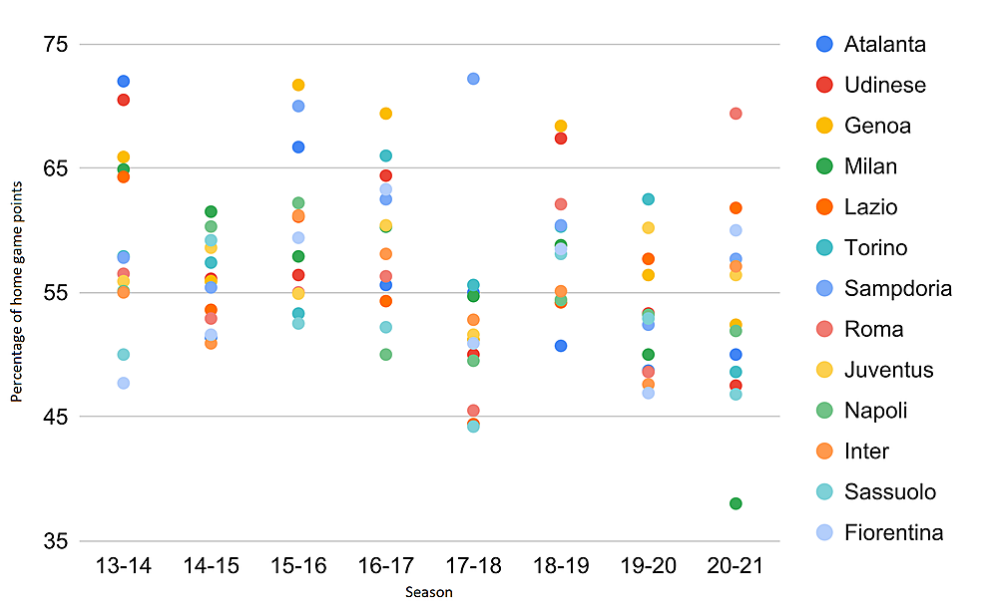
Percentage of home game points of teams that have never been relegated to Serie B in the 2013-2021 interval. Next to each marker, the ranking position reached by the club at the end of the season is shown.

Let us now consider only the clubs that have not been relegated to the lower league (Serie B) from 2013 to 2021. Figure 3 shows two pieces of evidence: (i) before COVID-19, one to three teams exceeded 65% of HGPs, while, after COVID-19, such interval was reduced from zero to one; and (ii) before COVID-19, the positions reached by teams that exceeded 65% of HGPs ranged from 9 to 17, while, after COVID-19, the only team that exceeded the threshold finished seventh. The club most affected by the anti-pandemic restrictions was AC Milan, which reached an absolute minimum of 38% of HGPs.

Referees

As shown in Table 4, penalties per game against home teams have increased substantially in the last two seasons, reaching figures closer to the ideal 50%. Indeed, although fouls and yellow cards have also approached that threshold (2.8 ≤ Δ% ≤ 4.3, 1.8 ≤ t ≤ 2.2), penalties against home teams have increased by 30%. However, we also point out that the number of red cards against home teams only increased slightly.

**Table 4:** Referee cumulative statistics from 2013 to 2021 in Serie A. The values shown correspond to the percentages of fouls, penalties, and cards whistled against the home teams. p.g. = per game; SEM = standard error of the mean.

Seasons	% Fouls	% Penalties p.g.	% Yellow cards	% Red cards
2013-2014	50.0	26.1	48.2	45.8
2014-2015	48.6	56.1	47.4	44.1
2015-2016	49.0	35.6	46.8	40.6
2016-2017	50.6	39.0	47.0	37.1
2017-2018	47.1	44.1	47.2	40.7
2018-2019	48.1	35.4	46.5	43.3
2019-2020	50.8	49.1	48.3	45.1
2020-2021	49.7	53.6	50.1	40.6
Average 2013-2019	48.9	39.4	47.2	41.9
SEM	0.5	4.1	0.2	1.3
Average 2019-2021	50.3	51.4	49.2	42.9
SEM	0.5	2.3	0.9	2.3
Δ%	2.8	30.4	4.3	2.2
t	1.8	2.6	2.2	0.4

## Discussion

Given the absence of an audience in the stadiums for long periods in the last two seasons of Serie A - the highest football league in Italy - we aimed to evaluate the conditioning of home support on both players and referees. The results we have found confirm the substantial role of home advantage. By comparing the last two seasons to the previous six (from 2013 to 2021), we observed two main findings: (f1) the average of points collected in home matches has significantly diminished, going from 58.1 (2013-2019) to 53.5% (2019-2021); and (f2) the number of penalties awarded against home teams has substantially increased, reaching the ideal 50%. Furthermore, two other pieces of evidence testify to a profound change in the physiognomy of the championship: indeed (f3) the negative correlation between the percentage of home points and total points collected has vanished; and (f4) the number of successful passes increased slightly, reaching two all-time highs of the series.

F1 shows that the public factor plausibly accounted for between 4% and 5% of the home advantage, while the remaining 3-4 percentage points can be linked to other factors. This result gives credence to the theory of Arboix-Alió et al., whereby difficulties due to away-team travels and unfamiliarity with the opponent's playing field can also be decisive [5]. At the same time, f2 suggests that the home crowd has a very pronounced potential deterrent effect on the referees' direction. From 2013 to 2019, penalties per game awarded against home teams made up about 39% of the total, while in the last two seasons, it reached 51%. This fact is particularly relevant considering that, during the 2017-2018 season, the so-called "video assistant referee" (VAR) was introduced in Serie A [24]. Specifically, VAR is a system created to guarantee impartiality in refereeing decisions based on multiple control of doubtful situations. Therefore, two inclusive hypotheses are possible: (i) referees and VAR teams were influenced by home crowds in awarding decisions against home teams [25-27]; and (ii) since the home teams lost a relevant fraction of the home advantage, the number of fouls in the penalty area has increased due to the lower concentration of home players and/or the greater conviction of the opponents [4]. Significant changes were also detected for other arbitration variables, such as the number of fouls and yellow cards against home footballers; however, percentage increases remained limited (from 3% to 4%), especially if compared to the number of penalties assigned. Significant changes were also detected for other arbitration variables, such as the number of fouls and yellow cards against home footballers; however, percentage increases remained limited (from 3% to 4%), especially if compared to the number of penalties assigned. Conversely and surprisingly, the increase in the number of red cards was only 2%. F3 highlights that, on average, the teams that collect high percentages of home points occupy a worse position in the standings. The phenomenon is due to the fact that a competitive team also manages to collect a large number of points in away matches. However, during COVID-19, this Serie A feature faded, reaching correlations close to 0. Hence, the absence of the audience seems to have heavily influenced the physiognomy of Serie A. Finally, the increase of successful passes detected in f4 is plausibly due to the decrease in the athletes’ stress concerning the absence of pressing cheer and noise. In particular, the above interpretation is consistent with the findings of Nicholls et al. [28]. Nonetheless, although statistically significant, the above change was moderate (from 1% to 5% depending on the seasons considered). Furthermore, not all the scientific literature agrees on this topic, for example, Cao et al. found that the performance of basketball players did not depend on the size of the crowd [29]. These results confirm the complex diversity of factors affecting concentration and mental state in sports performance. Indeed, individual sports differ from team sports because of the dynamics established within the group, specifically, the processing of stressful situations by the individual also depends on the perception and reaction of the teammates [30]. Moreover, the variability of contrasting findings concerning team sports discussed in the introduction underlines the importance of weighing the evidence on the period, the discipline, and the dimension of the investigated sample. For these reasons, the inference process requires strong theoretical bases to support it, and mere statistics can be exploited as a complementary tool only if based on clinical and psychological evidence; otherwise, no causal explanations can be asserted.

Strengths

To the best of the authors' knowledge, this is the first study that exploits a historical comparison via t-test and correlational analysis to investigate the impact of COVID-19 restrictive measures on Serie A. In particular, our findings provide evidence of a profound and significant change in many aspects of the championship, like home performance, refereeing, leaderboards structure, and competitiveness. Furthermore, targeting hypotheses decreases the likelihood of finding spurious correlations. Finally, this work proposes a simple but effective framework for statistically examining this type of phenomenon, even in fields outside the sport.

Limitations

This paper has some limitations to consider. First, we assumed that in the absence of extraordinary events such as COVID-19, the seasons 2019-2020 and 2020-2021 would have followed the trend of the previous six seasons. Second, statistical comparison alone does not provide causal explanations of the phenomena. However, the relevance of this point has been weakened thanks to the discussion of psychological theories supporting our findings. Third, the impact of the pandemic was not limited to preventing crowds from entering stadiums but may also have extended to the mental state of referees and players. Finally, the analysis is confined to Serie A only. 

## Conclusions

This research provides statistical evidence in favor of the relevance of home advantage in the top football league in Italy, such as Serie A. During the anti-COVID-19 restrictive measures - which prevented public access to the stadiums - a net reduction in the points collected by the teams in home matches was detected. In addition, the number of penalties awarded against home teams has increased significantly, approaching the ideal 50%. Since there are valid psychological reasons in the literature to support the crowd’s impact on sports and refereeing performance, it is plausible that our findings are causally related to the absence of cheering. Finally, as the averages of points collected at home remained far from 50%, our results suggest that a non-negligible part of the home advantage in Serie A is linked to factors independent of the audience. Future research can deepen the above phenomena from a theoretical-psychological point of view.

## Figures and Tables

**Figure 4:**
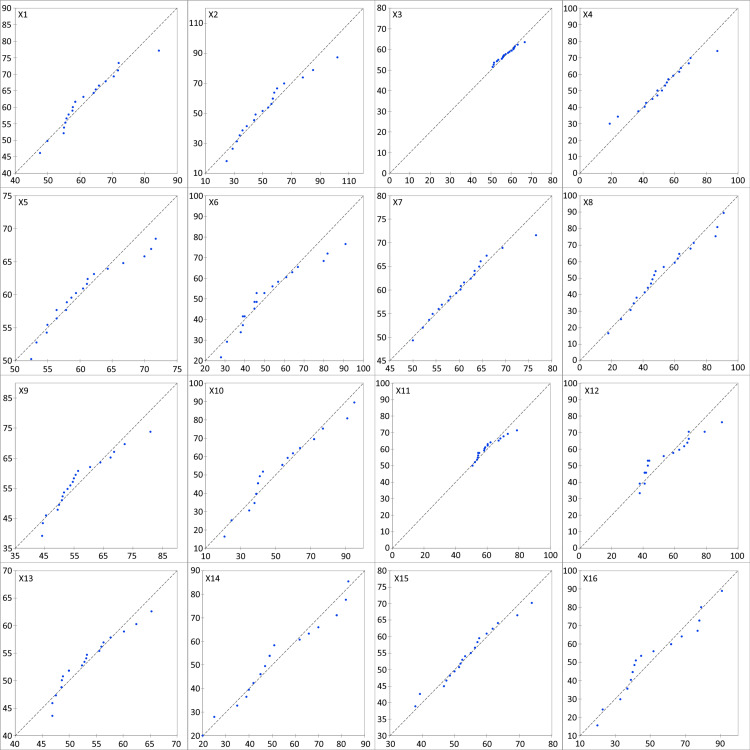
Percentage of home points and total points Q-Q plots from 2013 to 2021. X1 = 2013-2014 percentage of home points and X2 = 2013-2014 total points. The remaining variables follow the same logic until the 2020-2021 season.

**Figure 5:**
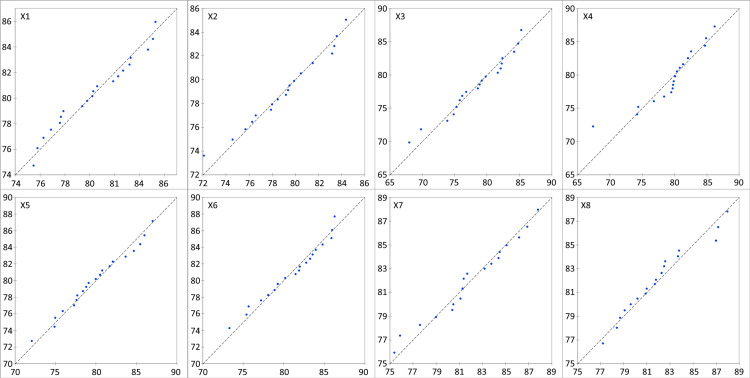
Percentage of successful passages Q-Q plots from 2013 to 2021. X1 = 2013-2014 percentage of successful passages. The remaining variables follow the same logic until the 2020-2021 season.

**Figure 6:**
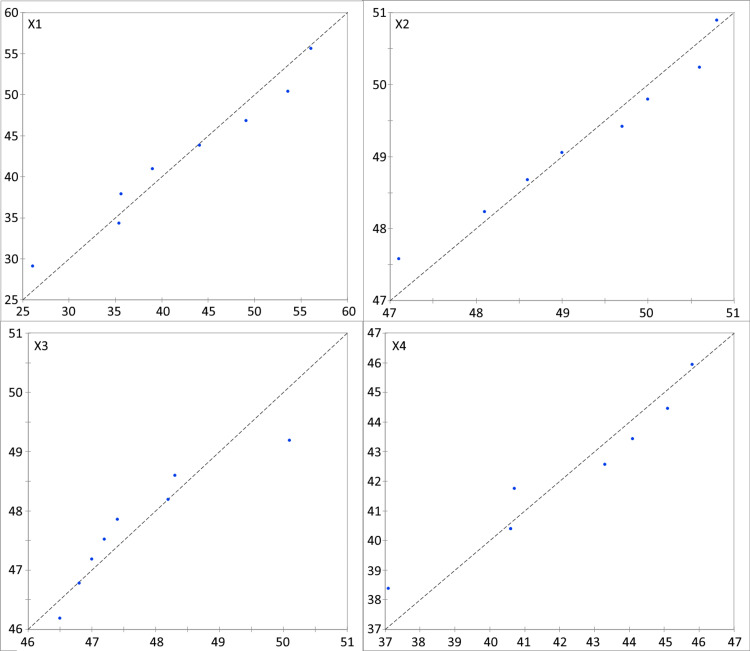
Referees statistics Q-Q plots from 2013 to 2021. X1 = percentage of penalties per game, X2 = percentage of fouls, X3 = percentage of yellow cards, and X4 = percentage of red cards.

## Appendices

Percentage of home points and total points normality test

The datasets are Gaussian as evidenced by Q-Q plots, Shapiro-Wilk P-values, and the correlations shown in Table 2 (Figure 4).

Shapiro-Wilk P-values vector (from X1 to X16): 0.238180, 0.205316, 0.754261, 0.995589, 0.622510, 0.109688, 0.737338, 0.577750, 0.0678510, 0.256432, 0.666476, 0.397968, 0.0767897, 0.252652, 0.882368, and 0.138040.

The average percentage of successful passage normality test

The datasets are Gaussian as evidenced by Q-Q plots and Shapiro-Wilk P-values (Figure 5).

Shapiro-Wilk P-values vector (from X1 to X8): 0.339839, 0.858150, 0.499251, 0.0520889, 0.910040, 0.561944, 0.670625, and 0.241446.

Referees statistics normality test

The datasets are Gaussian as evidenced by Q-Q plots and Shapiro-Wilk P-values (Figure 6).

Shapiro-Wilk P-values vector (from X1 to X4): 0.907110, 0.906146, 0.177491, and 0.581623.
